# The complete mitochondrial genome of the freshwater crab *Neilupotamon xinganense* (Decapoda: Brachyura: Potamoidea)

**DOI:** 10.1080/23802359.2020.1781570

**Published:** 2020-06-22

**Authors:** Qi-Hong Tan, Chun-Chao Zhu, Xiao-Juan Zhou, Shu-Xin Xu, Li-Yan-Yang Zhang, Lin-bo Shi, Jie-Xin Zou

**Affiliations:** a Research Laboratory of Freshwater Crustacean Decapoda and Paragonimus, School of Basic Medical Sciences, Nanchang University, Nanchang, China; b Key Laboratory of Poyang Lake Environment and Resource Utilization, Ministry of Education, Nanchang University, Nanchang, China

**Keywords:** Brachyuran, complete mitochondrial genome, phylogenetic, *Neilupotamon xinganense*

## Abstract

In this study, we first obtained the complete mitochondrial genome of *Neilupotamon xinganense* (Decapoda: Brachyura: Potamoidea). The genome is 16,965 bp in length and typically consists of 37 genes (13 protein-coding genes, 22 tRNAs genes, two rRNAs genes, and one putative control region). In addition, the mitogenome has 20 non-coding regions ranging from 1 to 683 bp in length. This study provides DNA data for further researches on population genetics and phylogenetics.

The genus *Neilupotamon* is endemic to mainland China, including *N. papilionaceum, N. physalisum, N. sinense,* and *N. xinganense.* The complete mitochondrial genome of *N. sinense* had been reported (Zhang et al. 2020). In this study, we report for the first time the complete mitochondrial genome of *N. xinganense*.

An adult specimen of *N. xinganense* was collected from Dayuan Village, Zhongfeng Town, Ziyuan County, Guilin City, Guangxi Province, China in 2018 (N25.9224°, E110.6572°). The sample has been deposited in the Laboratory Specimen Library of Freshwater Crustacean Decapoda and Paragonimus, School of Basic Medical Sciences, Nanchang University, Nanchang, Jiangxi, China, and National Parasite Germplasm Resources Specimen Library of China with a catalog number of NCUMCP4068. The sample was stored in 95% ethanol prior to extraction at room temperature before sequence analyses. Genomic DNA extraction, sequencing, gene annotation, and phylogenetic analyses were performed according to the method described by Plazzi et al. (2013). The Bayesian Inference (BI) method was performed using MrBayes vers. 3.2 (Ronquist et al. 2012), with best model GTR + I + G selected by jModelTest vers.2.1.7. The maximum-likelihood (ML) method was performed using MEGA 6 (Tamura et al. 2013).

The complete genome of *N. xinganense* is 16,965 bp in length (GenBank accession number: MN117718) and contains 13 protein-coding genes (PCGs), 22 tRNA genes, two rRNA genes, and one control region (CR) as the typical metazoan mitochondrial genome. However, in *N. xinganense*, we can only clearly annotate the location of 21 kinds of tRNA, the location of *tRNA^Ile^* is still in doubt. We try to search for traces of missing tRNA by inferring the evolutionary history of gene structure rearrangement. By comparing with the gene sequence of relative species, we can basically determine that the location of *tRNA^Ile^* should be located in the 86 bp intergenic region between *12S rRNA* gene and *tRNA^Met^*, and roughly determine that 64 bp is a special *tRNA^Ile^*, and its anticodon is also transformed from the normal GAT to TAT.

Among the 37 genes, 23 genes are encoded by H-strand and 14 genes are encoded by L-strand. The total length of coding gene is 14,738 bp, and the total length of non-coding region is 2227 bp. There are 20 non-coding regions with a length of 1–683 bp, the longest non-coding region is between *tRNA^Gln^* and *COX2*. There are seven overlapped regions with a total length of 21 bp and a length of 1–7 bp, among which the longest overlapped region is between *ND4* and *ND4L*. Among 13 protein-coding genes (PCGs), *ND1*, *ND4*, *ND4L,* and *ND5* are encoded by L-strand, and the other nine are encoded by H-strand. The start codon of *COX1*, *COX2*, *COX3*, *ATP8*, *ND2*, *ND3*, *ND4L*, *ND5,* and *CYTB* is ATG, the start codon of *ATP6*, *ND1*, and *ND6* is ATA, and the start codon of *ND4* is GTG. For stop codon, except *ND5* is TAT and *COX2* is incomplete T––, all the other genes are TAA. The average A + T content of PCGs is 64.93%. *ND4L* has the highest A + T content of 68.65%, *CYTB* has the lowest A + T content of 60.16%.

The length of 22 tRNA genes is between 62 bp (*tRNA^Cys^*) and 72 bp (*tRNA^Val^*). Fourteen tRNA genes are encoded by H-strand and the rest by L-strand. All tRNA genes except for *tRNA^Ser(AGN)^* and *tRNA^Ile^* showed typical cloverleaf structure. The 16S rRNA gene and 12S rRNA gene are encoded by L-strand, with length of 1330 bp and 826 bp respectively. The *16S rRNA* gene is located between *tRNA ^Leu(TAG)^* and *tRNA^Val^*, whereas the *12S rRNA* gene is located between *CYTB* and *tRNA^Ile^*. The longest non-coding region, the control region, lies between *COX2* and *tRNA^Gln^*, with a length of 683 bp and the A + T content of 72.18%.

The phylogenetic position of *N. xinganense* in mitogenome relative to other Brachyuran mitogenomes is determined by applying the BI and ML methods on 13 PCGs (Figure 1). Phylogenetic analysis showed that the two species *Bottapotamon lingchuanense* and *N. xinganense*, which have unique gene sequence, gather into one branch, and form sister branches with the evolutionary branch composed of four species of *Sinopotamon* and *Longpotamon*.

**Figure 1.**
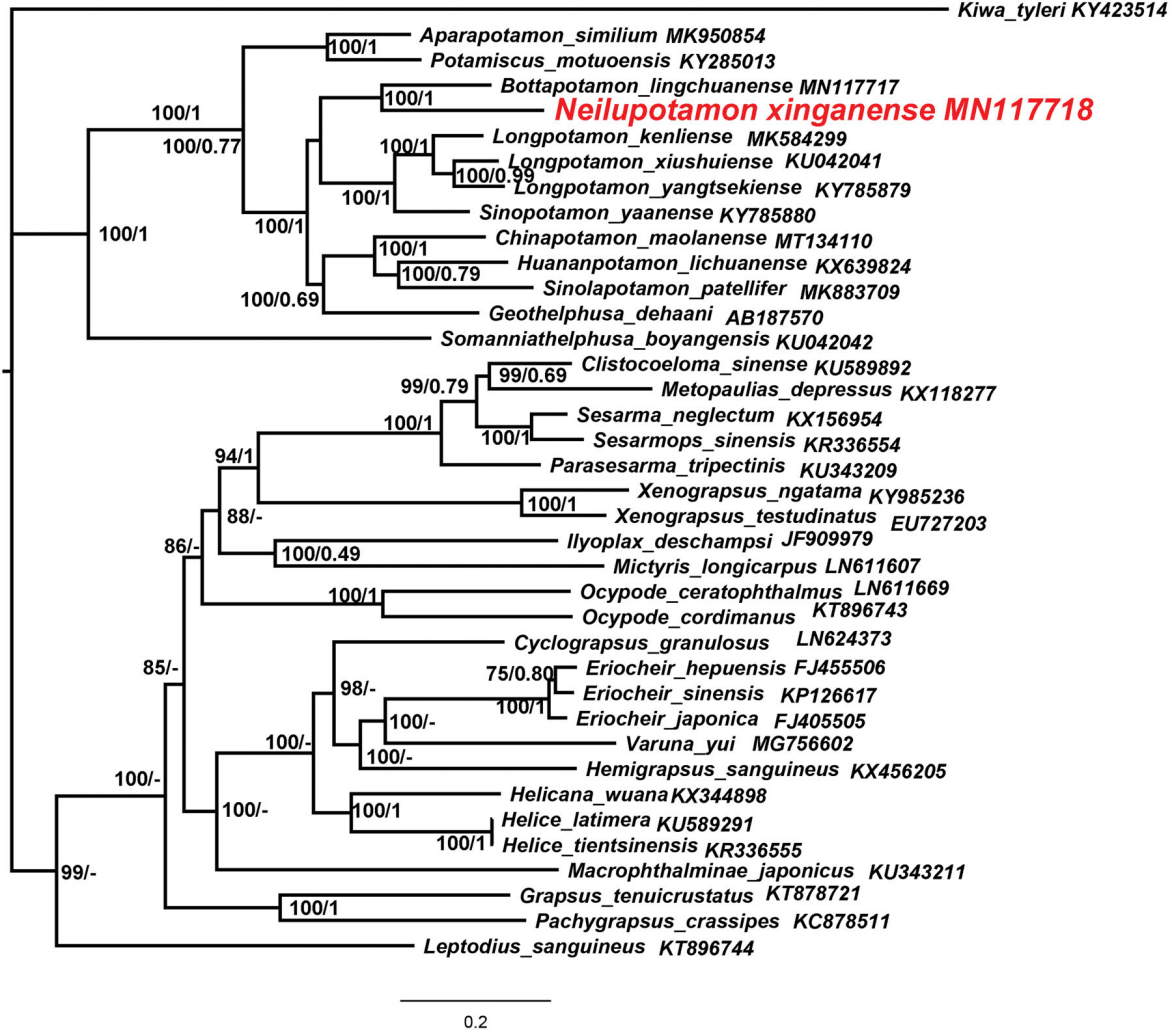
Phylogenetic maximum-likelihood (ML) tree of *Neilupotamon xinganense* and related brachyurans based on 13 PCGs nucleotide sequences from the mitochondrial genome. *Kiwa tyleri* serves as the outgroup. The numbers at the internodes are Bayesian inference (BI) bootstrap proportions and ML posterior proportions. The differences between the ML and BI trees are indicated by ‘–’. The scale bars represent genetic distance.

## Data Availability

The data that support the findings of this study are openly available in GenBank of NCBI at https://www.ncbi.nlm.nih.gov, reference number MN117718.
